# Associations between the duration of active commuting to school and academic achievement in rural Chilean adolescents

**DOI:** 10.1186/s12199-017-0628-5

**Published:** 2017-04-04

**Authors:** Antonio García-Hermoso, Jose M. Saavedra, Jordi Olloquequi, Robinson Ramírez-Vélez

**Affiliations:** 1 Laboratorio de Ciencias de la Actividad Física, el Deporte y la Salud, Facultad de Ciencias Médicas, Universidad de Santiago de Chile, USACH, Santiago de Chile, Chile; 2 Facultad de Ciencias de la Educación, Universidad San Sebastián, Santiago de Chile, Chile; 3 Physical Activity, Physical Education, Sport and Health Research Centre, Sports Science Department, School of Science and Engineering, Reykjavik University, Reykjavik, Iceland; 4 Instituto de Ciencias Biomédicas, Facultad de Ciencias de la Salud, Universidad Autónoma de Chile, Talca, Chile; 5 Centro de Estudios para la Medición de la Actividad Física «CEMA». Escuela de Medicina y Ciencias de la Salud, Universidad del Rosario, Cra. 24 No. 63C - 69, Bogotá, DC Colombia

**Keywords:** Active travel, Physical activity, Walking, School performance

## Abstract

**Background:**

Habitual active commuting to school may be positively associated with academic achievement. The aim of this study was to examine the relationship between duration of walking or otherwise actively commuting to school and academic achievement.

**Methods:**

This cross-sectional study included 389 adolescents from seven rural schools (12–13 years). Mode and duration of active commuting to school (use of active means such as walking or biking to and from school) and screen time were self-reported. Academic achievement was determined by the outcome in basic grades (language and mathematics).

**Results:**

Active commuting to school was not associated with higher scores in any grades after adjustment for potential confounders. No evidence was found of interactions between gender and academic achievement, but there was interaction with duration of walking (<30 min, 30–60 min, and >60 min). Adjusted binary logistic regression analysis suggested that adolescents who spent between 30 and 60 min actively commuting were more likely to obtain high academic achievement (language and mathematics).

**Conclusions:**

Thirty to 60 min of ACS may have a positive influence on academic achievement in adolescents, so, it is necessary to make recommendations for the children to walk from and/or to school. This could help society to recognize the relevance of physical activity to health as well as to academic performance.

## Background

Cultural shifts and changes in home and neighbourhood environments discourage physical activity (PA) in young people [1]. Active commuting to school (ACS), defined as the use of active means such as walking or biking to and from school, is an inexpensive form of PA that can be integrated into adolescents’ routines [2]. It has been argued that if sufficient intensity is achieved, ACS could lead to an increase in cardiovascular fitness [3] and is associated with a healthier body composition and metabolic profile [4]. Several studies suggest that adolescents who actively commute to and from school can accumulate an additional 20 min of moderate to vigorous PA across the whole day, compared to adolescents who use passive transport [5].

The evidence presented in a recent review suggests that health markers (including physical activity, nutrition, and body composition) affect the structure and function of the hippocampus, a structure known to be critical for successful learning and remembering [6]. Given that studies suggest that PA has positive effects on academic achievement [7] and cognitive performance in adolescents [8], habitual ACS may be positively associated with these as previously the evidence have suggested [9]. These works have studied the relationship between ACS and academic achievement in an adolescent population. For example, Martinez-Gomez et al. [9] reported a positive association between ACS and cognitive performance in urban Spanish adolescent girls, especially in those who reported more than 15 min of ACS per day compared to girls who spent fewer than 15 min. However, this study [9] evaluated cognitive performance with SRA Test of Educational Ability which measured in the general way verbal (command of language), numeric (speed and precision in performing operations with numbers and quantitative concepts), and reasoning (the ability to find logical ordination criteria in sets of numbers, figures, or letters) abilities.

Youth from rural areas and small cities were more active than urban children, although the differences were small to moderate [10]. Regarding ACS, evidence suggests that youth who live in rural areas are less likely to actively commute to school than those living in urban areas [11], which may be related to fewer pedestrian infrastructures, longer commuting routes, and poorer access to public transport. Despite the growing interest in active travel to and from school, none studies have explored the duration of walking ACS and its relationship with academic achievement from rural areas. To the best of our knowledge, the ACS-academic achievement relationship has not been assessed in a Latin American country at the rural population level. Thus, the aim of this study was to examine the relationship between duration of walking ACS and academic achievement in rural Chilean adolescents. We hypothesize that in rural areas the duration of walking ACS has a direct relationship with the academic achievement, i.e. big duration, big academic achievement.

## Methods

### Study design and participants

Data collection took place between March and June 2014. All students [*n* = 454] from seventh grade schools in the Maule region (Chile) were invited to participate. In seventh grade the students meet their thirteenth in the academic year. The schools were selected for accessibility (convenience sampling consisted of selecting schools that could most easily or willingly participate in this study). Subjects were excluded if they had special educational needs [learning difficulties and/or learning disabilities] or had any type of dysfunction limiting their PA (any disease or problem). Physical education teachers provided this information. Also, due to the small number of adolescents (*n* = 6) who commuted actively to or from school by cycling, they were excluded. Thus, we analyzed only subjects who commuted actively to or from school by walking. Finally, a sample of 389 adolescent students (12–13 years, 86% of invited), 196 boys and 193 girls, agreed to participate in the study. The study protocol was approved by the (*blinded for purposes of review*) Ethics Committee and complied with the principles of the Declaration of Helsinki. The study was conducted according to ethical standards in sport and exercise science research [12]. A letter was sent to parents of all adolescents in the seventh grade. It invited them to a meeting where the objectives were explained, after which they signed the informed consent for the participation of their adolescents in the study.

## Mode and duration of commuting to school

The mode of commuting to and from school was measured by the self-reported questionnaire. Two questions were asked about the mode and duration of commuting to school: (1) “How do you usually travel from home to school and from school to home?” and (2) “How long does it usually take you to travel from home to school or from school to home?” Response options to the first question were walking, biking, bus, car/motorcycle, and others. Response options to the second question included 15 min or shorter, from 15 to 30 min, from 30 to 60 min, and longer than 60 min. Responses were categorized as: ACS ≤30 min, ACS 30–60 min, and ACS > 60 min. If subjects reported at least one of the trips as active [i.e. to or from school], they were included in the active commuting group (ACS group). In contrast, if subjects not reported at least one of the trips as active, they were included in the non-active commuting group (non-ACS group).

Previous studies have demonstrated evidence of the reliability and validity for similar questions [13].

### Academic achievement

Academic achievement was assessed using the students’ grades in mathematics and language using a standard process within Chile public schools (same curriculum and contents). The grades were collected from the schools’ official records at four time points in the first semester (March, April, May and June, 2014). In Chilean elementary schools, student grades range from 1 (worst) to 7 (best), classified as: very poor = 1.0–1.9; poor = 2.0–2.9; below average = 3.0–3.9; average = 4.0–4.9; good = 5.0–5.9; very good = 6.0–7.0. The average score was calculated, determining high academic achievement with a grade above or equal to 5.0 (good and very good scores) [14].

### Potential confounders

At the beginning of each potential confounder, the reason for its inclusion in the study has been explained.

#### Anthropometry

Several studies have reported negative associations between obesity and school achievement [14]. For that reason, the body height and weight were measured with the adolescents having bare feet and wearing light underclothes, using an electronic scale (Seca, Berlin, Germany). Body mass index (BMI = kg/m^2^) and height for age were evaluated and *z*-scores were obtained according to the Centers for Disease Control and Prevention [15] references. Weight status was defined as follows: underweight (*z*-score < −1 SD), normal weight (*z*-score from −1 SD to 1 SD), at risk of obesity (*z*-score from >1 s to <2 SD) and obese (*z*-score ≥2 SD).

#### Screen time

This parameter is inversely related to academic achievement [14]. So, three questions from the *Health Behavior in School-Aged Children* study were used [16] regarding daily television, videogame and computer use: ‘About how many hours a day do you usually: (i) watch television; (ii) play computer or video games; (iii) use a computer [for purposes other than playing games—for example, emailing, chatting, or surfing the internet or doing homework] in your free time?’ Daily screen time averages were calculated by adding the answers to these three questions together. Finally, screen time was dichotomized as recommended (<2 h/d) and excessive (≥2 h/d), based on the American Academy of Pediatrics’ international guidance on limiting paediatric screen time [17].

#### Physical activity

A recent review suggests that PA is positively related to academic achievement [18]. For that, the PA was measured with the self-administered Spanish version of the *Physical Activity Questionnaire for Adolescents* (PAQ-A) [19]. The questionnaire was designed to assess adolescents’ levels of moderate and vigorous PA. Adolescents were asked to quantify their PA levels during their spare time in the previous week. Nine items scored on a five-point Likert scale were averaged to derive an overall PA score ranging from one to five (higher scores indicating higher levels of PA). Finally, physical activity was dichotomized as high (four quartile) and low-medium (first to third quartile).

#### Maternal education

The majority of the literature on parents’ education pertains to the direct and positive influence on achievement for children and youths [20]. Mothers completed a questionnaire about their highest level of education and were dichotomized as having an education level below the university level or an education at or above the university [post high school] level.

#### Socioeconomic status

There is evidence indicating that socioeconomic status (SES) is one of the most important determinants of childhood school achievement [21]. Therefore the SES was obtained using a scale based on Graffar’s modified method [22], which considers items such as schooling, job held by the head of the household and characteristics of the house, taking into account three categories (high, medium, and low SES).

#### Neonatal characteristics

Birth weight is associated with cognitive impairments persisting into childhood [23]. Therefore, the birth weight (kg) was reported by parents and dichotomized as low birth weight (≤2.500 g) and normal birth weight (>2.500 g) [24].

### Data analysis

Descriptive statistics were performed to characterize the sample. The continuous variables were expressed as the mean and standard deviation and frequency distribution for categorical data. Statistical normality was tested using the Kolmogorov-Smirnov test. Due to their skewed distribution, the language and mathematics variables were natural log-transformed. To aid interpretation, data were back-transformed from the log scale for presentation in the results to examine the associations between mode of commuting to school (non-ACS or ACS) and academic achievement. The analysis of covariance was conducted after adjusting for gender, weight status, screen time, PA, maternal education, SES, school, and birth weight. The interaction between gender and ACS categories (i.e., duration of ACS) was performed by means of a two-way ANOVA. This interaction term was not significant (*data not shown*) and so there was no stratification by gender. ANCOVA models were estimated to test differences in academic by ACS categories adjusted for sex, weight status, screen time, PA, maternal education, SES, school, and birth weight. Pairwise post-hoc comparisons were examined using the Bonferroni test. Finally, a model was considered using high academic achievement (score ≥ 5.0) as the dependent variable and the primary explanatory variable was the duration of ACS category. The model was adjusted variables abovementioned. Variable selection for the logistic regression models was also guided by the bivariate analyses. The Hosmer-Lemeshow goodness-of-fit test was used to assess the fit of the multiple logistic models. Data analysis was performed using IBM SPSS Statistics (version 22; SPSS, Inc., Chicago, IL, USA). A *p*-value of ≤0.05 denoted statistical significance.

## Results

Table 1 shows the adolescents’ characteristics. The sample SES was predominantly (68.6%) medium. Around 23% of the students made at least one trip (to or from school) by active means. There were no differences in academic achievement (language and mathematics) between walking ACS categories (non-ACS and ACS) adjusted for sex, weight status, screen time, PA, maternal education, SES and birth weight: language (5.02 ± 0.43, non-ACS vs 5.12 ± 0.45, ACS; *p* = 0.836) and mathematics (5.14 ± 0.42, non-ACS vs 5.58 ± 0.65, ACS; *p* = 0.677).

**Table 1:** Descriptive characteristics for the participants in this study (*n* = 389)

Girls, *n* (%)	193 (49.6)
Age, years	12.0 ± 0.6
Weight status ^a^	
Underweight, *n* (%)	4 (1.0)
Normal, *n* (%)	172 (44.2)
Overweight, *n* (%)	115 (29.6)
Obesity, *n* (%)	98 (25.2)
Birth weight	
Low birth weight (≤2500 g), *n* (%)	20 (5.1)
Self-reported physical activity	
High ^b^	72 (18.5)
Sedentary behaviour	
Exceeding recommended limits ^c^, *n* (%)	266 (68.4)
Mother’s education	
Level university, *n* (%)	57 (14.7)
SES	
Low, *n* (%)	52 (13.4)
Medium, *n* (%)	267 (68.6)
High, *n* (%)	70 (18.0)
Academic achievement	
Language (1–7)	5.0 ± 0.9
High academic achievement, *n* (%)	217 (57.7)
Mathematics (1–7)	5.3 ± 0.6
High academic achievement, *n* (%)	238 (61.3)
Means of transport	
Public transport, *n* (%)	148 (38.0)
Car/Motorbike, *n* (%)	152 (39.1)
Frequency	
Active transport to school, *n* (%)	89 (22.9)
Active transport from school, *n* (%)	111 (28.5)
Duration of ACS ^d^	
Non-ACS, *n* (%)	273 (70.2)
ACS ≤30 min, *n* (%)	55 (14.1)
ACS 30–60 min, *n* (%)	40 (10.3)
ACS ≥60 min, *n* (%)	21 (5.4)

Values are means (standard deviations ± SD) and number and proportions (%) for categorical data. ACS, active commuting to school; BMI, body mass index; SES, socio economic status. ^a^, Weight status defined as follows: Underweight (z-score < −1 s), Normal weight (z-score from −1 s to 1 s), Obesity risk (z-score from 1 s to 2 s) and Obesity (z-score >2 s); ^b^, Physical activity ≥2.09; ^c^, ≥2 h/day of screen time; ^d^, duration of walking active commuting to school and/or from school

**Fig. 1:**
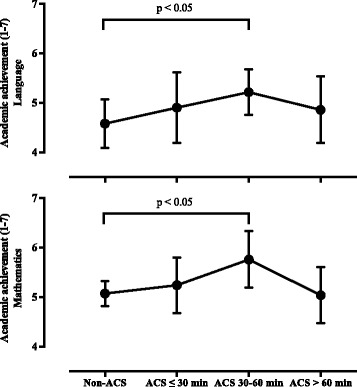
Mean differences in academic achievement by categories of active commuting to school. ACS, active commuting to school

Figure 1 shows mean differences in academic achievement by categories of ACS. Academic attainment was higher in adolescents with 30 to 60 min of ACS than non-commuters in language (*p* = 0.016) and mathematics (*p* = 0.031)

Table 2 shows the OR of the relationship between academic achievement and duration of ACS. In both grades, adjusted analysis suggests that students with 30 to 60 min of ACS were more likely to have a better academic achievement than non-commuters (language, OR = 3.53, 95% CI, 1.12 to 4.37; *p* = 0.003; mathematics, OR = 2.19, 95% CI, 1.06 to 5.05; *p* = 0.028). The Hosmer-Lemeshow goodness-of-fit test suggests that each of the models fit reasonably well, with *p* values 0.818 and 0.933 in language and mathematics, respectively.

**Table 2:** Logistic regression analyses for duration of walking ACS associated with academic achievement in Chilean adolescents, after adjusting for sex, weight status, birth weight, physical activity, screen time, maternal education, and SES

	Language		Mathematics	
	OR (95% CI)	*p*	OR (95% CI)	*p*
Sex				
Girls	1.00		1.00	
Boys	2.09 (1.19 to 3.67)	0.010	1.19 (0.71 to 2.01)	0.506
Weight status				
Normoweight	1.00		1.00	
Overweight + obesity	0.64 (0.40 to 0.95)	0.023	0.77 (0.46 to 1.31)	0.343
Birth weight				
> 2500 g	1.00		1.00	
≤ 2500 g	0.22 (0.04 to 1.24)	0.085	0.88 (0.34 to 2.28)	0.869
Self-reported physical activity				
Low-Medium	1.00		1.00	
High	1.45 (0.76 to 2.77)	0.261	1.35 (0.71 to 2.56)	0.353
Screen time recommendation				
< 2 h/day	1.00		1.00	
≥ 2 h/day	0.98 (0.59 to 1.65)	0.962	0.25 (0.13 to 0.50)	<0.001
Mother’s education				
Below university level	1.00		1.00	
University level	0.66 (0.30 to 1.43)	0.290	1.38 (0.67 to 2.83)	0.375
SES				
Low, *n* (%)	1.00		1.00	
Medium, *n* (%)	0.67 (0.19 to 2.43)	0.547	1.80 (0.45 to 7.30)	0.406
High, *n* (%)	1.38 (0.59 to 2.80)	0.433	1.08 (0.68 to 1.73)	0.737
Active commuting to school				
Non-ACS	1.00		1.00	
ACS ≤30 min	1.32 (0.72 to 2.41)	0.363	1.20 (0.69 to 2.07)	0.523
ACS 30-60 min	3.53 (1.12 to 3.37)	0.003	2.19 (1.06 to 5.05)	0.028
ACS ≥60 min	0.37 (0.06 to 2.21)	0.277	0.54 (0.12 to 2.48)	0.433
Likelihood ratio (Chi^2^)	116.18	<0.001	40.27	<0.001
Hosmer–Lemeshow	3.66	0.818	2.423	0.933
Correctly classified (%)	72.2		63.7	
Observations	389		389	

Although differences by sex, weight status, and screen time are not the main focus of this article, some comments should be made on their effect on the outcome variables. In Chile, the male students traditionally show better performance in the standardized tests, but in this study they were more likely to perform better in language, but not in mathematics. On the other hand, obese adolescents had worse grades than their counterparts with lower adiposity in language. Finally, adolescents who exceeded two hours of screen time daily had worse mathematics grades than adolescents who meet the screen time recommendations.

## Discussion

The aim of this cross-sectional study was to analyse the relationship between duration of walking ACS and academic achievement in rural Chilean adolescents. The results suggest that adolescents who spent 30 to 60 min on ACS were more likely to achieve a high academic achievement compared to non-ACS, independent of potential confounders (sex, weight status, PA, screen time, maternal education, SES, school, and birth weight).

The ACS represented 23% of the total time spent moving per week, and in addition to the health benefits of PA, neuroscience evidence also suggests that PA may exert a positive effect on children’s cognitive functioning [functional brain changes], brain physiology (changes in regional brain volume are associated with increased peripheral brain-derived neurotrophic factor and memory) and academic achievement [25]. Also, a recent cross-sectional study does support a positive relationship between PA and mathematics at 16 years [7]. Studies have reported that adolescence is a sensitive period to stimulate cognitive function and improve learning and academic achievement; however, in Chile it is the period of life with the greatest decline in PA levels [26]. Therefore, ACS is an opportunity to increase daily PA levels and consequently cognitive performance at school. The results support that ACS, adjusted by potential confounders, is not associated with academic achievement in either boys or girls. A recent study observed that when combined with obesity, low-medium levels of PA and excessive screen time might be related to poor academic achievement [14]. In this sense, obesity is associated with poor executive functioning, which is critical for academic achievement [27]. On the other hand, evidence has confirmed our results and have not observed a relationship between ACS and academic achievement [28].

Questions regarding the appropriate dose of ACS required to produce optimal outcomes in cognitive performance and academic achievement remain unanswered. A long distance to school implies a decreased likelihood of adopting active transport practices [29] and a distance of approximately two kilometres is associated with the best PA outcomes related to active transport [30]. Regarding duration, Martinez-Gómez et al. [9] reported that adolescent girls who spent more than 15 min on ACS had better cognitive performance variables than those who spent less time (≤ 15 min) and non-commuters. The results suggested that adolescents who spent 30 to 60 min on ACS were more likely to obtain high academic achievement compared to non-ACS adolescents, independent of potential confounders including PA and weight status. This relationship would have an inverted U-shape, where both too little (≤ 30 min) and too much ACS (≥60 min) has a non-positive association with academic achievement. This fact could be consider a novel finding, indicating range where the ACS has relationship with academic achievement. In this way, 60 min or more could indicate long trip to school. This oblige to children wake-up too much early and, probably to have an insufficient sleep that has influence in academic achievement [31]. On the other hand, 30 min or less could be consider a short time to achieve improvement. Possibly, if this time was of “brisk” walking (nearer of “physical fitness” concept) could be have positive effects [32].

Generally, studies support that only vigorous physical activity might have a crucial role in improving cognitive function and memory [33]. However, intensity associated with this routine is usually light or moderate, and it was suggested that this type of intensity plays a role in cognitive performance [9, 28]. Therefore, the results combine with others stating that a longer duration of ACS may positively influence school achievement. The results also showed that adolescents who spent 60 min or more actively commuting to school were no more likely to obtain a high mean academic achievement. These results could be due to adolescents who walk for longer normally waking before others, which could influence school performance. In this vein, a recent study of Portuguese adolescents suggests that waking up early is a predictor of study methods, reading skills, motivation for study and overall performance [34]. In the same way, these students would arrive home more tired than others, and it could hinder their homework. Therefore, more prospective and experimental studies are needed to further elucidate the complex relationships between ACS and cognitive performance.

The study has several limitations. First, the study is a cross-sectional design, which does not allow for the drawing of conclusions on the causal direction of the associations. Second, the sample is not representative of the population, so it difficult to generalize the results across the Chilean adolescent population. However, the sample is relevant: it gives interesting information. Third, the ACS (mode and duration of commuting to school) and PA variables were self-reported and we have not assessed the distance to and from school; therefore, findings must be interpreted with caution. Fourth, only 23% of the students made at least one trip (to or from school) by active means, which limits the statistical power. Fifth, using school-based grades given by teachers is subject to bias. Finally, the intensity of the ACS was not evaluated and higher intensity level of ACS could be associated with higher grades [33].

## Conclusions

The results of this study suggest that rural adolescents who spent 30 to 60 min on ACS were more likely to achieve a high academic achievement (language and mathematics) compared to non-ACS adolescents. Therefore, efforts to maintain and increase walking to school may be particularly relevant as this is likely to have a positive impact on adolescents’ school achievement. However, future researches in this field are necessary, especially intervention studies to increase PA and academic achievement.

## Abbreviations

ACS: Active commuting to school
PA: Physical activity
SES: Socioeconomic status
